# Nonfatal Assaults Among Persons Aged 10–24 Years — United States, 2001–2015

**DOI:** 10.15585/mmwr.mm6705a1

**Published:** 2018-02-09

**Authors:** Corinne F. David-Ferdon, Tadesse Haileyesus, Yang Liu, Thomas R. Simon, Marcie-jo Kresnow

**Affiliations:** ^1^Division of Violence Prevention, National Center for Injury Prevention and Control, CDC; ^2^Division of Analysis, Research, and Practice Integration, National Center for Injury Prevention and Control, CDC.

In 2015, persons aged 10–24 years who were treated for nonfatal assault injuries in emergency departments (EDs) in the United States accounted for 32% of the approximately 1.5 million patients of all ages that EDs treated for nonfatal assault injuries (*1*). CDC analyzed data from the National Electronic Injury Surveillance System–All Injury Program (NEISS-AIP) to examine 2001**–**2015 trends in nonfatal assault injuries among youths treated in EDs, by sex and age group, and to assess current rates by sex, age group, mechanism of injury, and disposition (*1*). Rates for 2001–2015 were significantly higher among males than among females and among young adults aged 20–24 years than among youths aged 10–14 and 15–19 years. During 2011–2015, rates declined for all groups. The 2015 rate among persons aged 10–24 years was 753.2 per 100,000 population, the lowest in the 15-year study period. Despite encouraging trends, the assault rate among young persons remains high. Rates in 2015 were higher among males, persons aged 20–24 years, and those who incurred intentional strike or hit injuries. Nearly one in 10 patients were admitted to the hospital, transferred to another hospital, or held for observation. Youth violence prevention strategies, including primary prevention approaches that build individual skills, strengthen family relationships, or connect young persons treated in EDs to immediate and ongoing support, can be implemented to decrease injuries and fatalities (*2*).

NEISS-AIP collects data from a nationally representative sample of EDs, using specific guidelines for recording the primary diagnosis and mechanism of all types of treated injuries. NEISS-AIP is operated by the U.S. Consumer Product Safety Commission in collaboration with CDC’s National Center for Injury Prevention and Control. Data are accessible using CDC’s Web-based Injury Statistics Query and Reporting System (*1*). The analysis was limited to patients treated for nonfatal assault injuries, which included injury resulting from an act of violence where physical force by one or more persons was involved and excluded injuries related to sexual assault. Data were stratified by calendar year, sex, and 5-year age group (10–14, 15–19, and 20–24 years). Data for 2015 were also stratified by mechanism of injury (struck by/against, cut/pierce, firearm, or other) and disposition (treated and released, transferred to another hospital, held for observation, left against medical advice, or left without being seen by physician). Annual injury rates (per 100,000 population) were computed overall and for the indicated strata. Joinpoint regression* was used to test the significance of trends from 2001 to 2015. Changes in the annual nonfatal assault rate among persons aged 10–24 years by sex and age group were examined. Annual percentage change (APC) estimates that were statistically significant (p<0.05) are presented to indicate the magnitude and direction of significant trends.

During 2001–2015, approximately 9.6 million persons aged 10–24 years were treated in EDs for nonfatal assault injuries, an average annual rate of 1,003.9 per 100,000 (Table). Rates were significantly higher among males (1,265.3 per 100,000) than among females (729.0). Rates were higher for young adults aged 20–24 years (1,376.5) than for persons aged 10–14 years (461.7) and 15–19 years (1,159.7). The overall nonfatal assault rate per 100,000 persons aged 10–24 declined during the 15-year study period from 1,179.7 in 2001 to 753.2 in 2015, the lowest rate in the study period (Figure 1). During 2011–2015, the overall nonfatal assault injury rate declined 27.5% (Table). During this period, rates for males and females declined 30.1% and 22.7%, respectively; the average annual percentage decrease was 8.5% for males and 7.4% for females (Figure 1). Also during 2011–2015, rates for persons aged 10–14, 15–19, and 20–24 years declined 35.5%, 30.6%, and 23.8%, respectively (Table). The injury rate declined 11.5% per year for persons aged 10–14 years, 9.2% for persons aged 15–19 years, and 5.6% for persons aged 20–24 years (Figure 2).

**TABLE:** Average annual rate of nonfatal assault injuries per 100,000 population among persons aged 10–24 years treated in hospital emergency departments, by sex and age group — United States, 2001–2015

Characteristic	No. of sample cases	National estimate* (%)	Average annual rate^†^ (95% CI)	No. of joinpoints	Joinpoint year range	APC	Rate^†^ range during joinpoint year	% reduction in rate during joinpoint year range
**Total**	**185,645**	**9,603,933 (100.0)**	**1,003.9 (805.0–1,202.8)**	**1**	**2001–2011**	**−1.4^§^**	**(1,179.7–1,039.0)**	**11.9**
					**2011–2015**	**−6.8^§^**	**(1,039.0–753.2)**	**27.5**
**Sex**								
Male	120,930	6,200,495 (64.6)	1,265.3 (1,003.6–1,527.1)	1	2001–2011	−1.2^§^	(1,476.8–1309.5)	11.3
					2011–2015	−8.5^§^	(1,309.5–914.9)	30.1
Female	64,687	3,401,887 (35.4)	729.0 (589.9–868.1)	2	2001–2008	−3.4^§^	(866.6–676.4)	21.9
					2008–2011	4.5	—	—
					2011–2015	−7.4^§^	(755.7–583.9)	22.7
**Age group (yrs)**								
10–14	34,132	1,447,593 (15.1)	461.7 (321.2–602.2)	2	2001–2008	−8.7^§^	(683.2–385.7)	43.5
					2008–2011	2.1	—	—
					2011–2015	−11.5^§^	(413.7–267.0)	35.5
15–19	74,267	3,724,730 (38.8)	1,159.7 (930.2–1,389.1)	1	2001–2011	−1.7^§^	(1,362.0–1,170.8)	14.0
					2011–2015	−9.2^§^	(1,170.8–813.1)	30.6
20–24	77,246	4,431,610 (46.1)	1,376.5 (1,132.3–1,620.7)	1	2001–2011	0.1	—	—
					2011–2015	−5.6^§^	(1,494.5–1,138.6)	23.8

**Abbreviations:** APC = annual percentage change; CI = confidence interval.

* Excludes sexual assault cases; includes assault cases with unknown sex. Estimates might not sum to total because of rounding.

^†^ Crude rate per 100,000 population.

^§^ Statistical significance of regression results (p<0.05).

**FIGURE 1:**
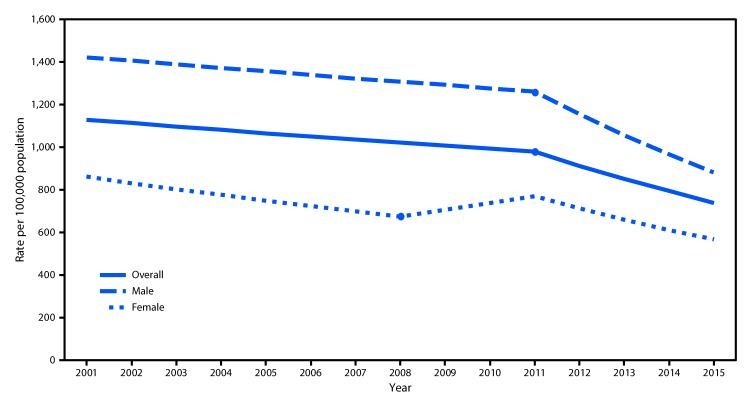
Nonfatal assault* injury rate among persons aged 10–24 years treated in hospital emergency departments, by sex — United States, 2001–2015^†^ * Excluding sexual assault. ^†^Joinpoint regression analysis was used to determine annual percentage change with statistically significant trend and significant joinpoints indicated (p<0.05).

**FIGURE 2:**
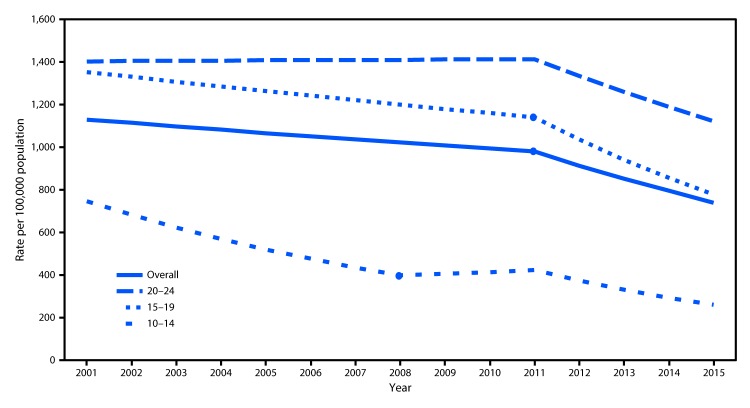
Nonfatal assault* injury rate among persons aged 10–24 years treated in hospital emergency departments, by age group — United States, 2001–2015^†^ * Excluding sexual assault. ^†^Joinpoint regression analysis was used to determine annual percentage change with statistically significant trend and significant joinpoints indicated (p<0.05).

In 2015, an estimated 485,610 persons aged 10–24 years were treated in EDs for nonfatal assault injuries. The rate of nonfatal assault injuries among persons aged 10–24 years was 914.9 per 100,000 for males and 583.9 for females; by age group, it was 267.0 per 100,000 for persons aged 10–14 years, 813.1 for persons aged 15–19 years, and 1,138.6 for persons aged 20–24 years.

Most persons aged 10–24 years treated in an ED for nonfatal assault injuries (81.2%) were treated for injuries related to being intentionally struck or hit. Other leading mechanisms of nonfatal injuries included being cut, stabbed, or pierced (8.1%), and having firearm-related injuries (5.7%). Most persons in this age range who visited an ED for assault injuries were treated and released (87.0%); 9.9% were hospitalized, transferred to another hospital, or held for observation; and 3.1% left the ED against medical advice or left without being seen by a physician.

## Discussion

For decades, young persons have represented a substantial proportion of patients receiving treatment in EDs for assault injuries. The findings in this report demonstrate that the rate of nonfatal assault injuries among persons aged 10–24 years has declined since 2001, with significant declines overall and by sex and age group since 2011. These encouraging declines are consistent with previous analyses and recent trends in youth violence (*3*,*4*). The declines might indicate increased implementation and beneficial effects of evidence-based prevention strategies that reach young persons (*2*,*5*). A number of primary prevention strategies have been shown to reduce the risk for and occurrence of youth violence, including school-based programs that build communication and problem-solving skills and family approaches that help caregivers set age-appropriate rules, monitor youth activities and relationships, and address other risk factors (e.g., childhood conduct problems and delinquency) (*2*).

The ED is an important implementation setting for prevention, in part because a large proportion of patients will experience a subsequent assault-related injury or premature death within a few years of a treated injury (*6*,*7*). For example, one study compared persons aged 14–24 years who sought treatment in the ED and reported substance use in the 6 months before the visit. Of the young persons who were seen initially for an assault-related injury, 36.7% were seen again for an assault-related injury within 24 months, compared with 22.4% of the young persons initially seen for other conditions (e.g., unintentional injury or illness) (*6*).

The implementation of brief ED interventions to reduce the continuation and escalation of violence is growing (*8*). These programs vary in design and duration but typically identify youths in the ED when they are examined for a violence-related injury. The programs are implemented by trained staff members (e.g., medical personnel, community service providers, and program outreach workers) who provide immediate and follow-up services to increase risk awareness, conflict resolution skills, and connection to community support (e.g., academic or vocational supports and mental health treatment). Research has shown that these programs have significant benefits, including sustained reductions in perpetration and victimization of peer violence (*9*). Evaluation of a specific program found that participants were 70% less likely than nonparticipating youths to be arrested for any offense during the 6 months after the program (*10*).

The findings of this report are subject to at least four limitations. First, injury rates are likely underestimates of the actual prevalence because data are limited to persons treated in EDs and do not include those who had injuries treated in other health care facilities (e.g., physician’s office or urgent care center) or those for whom no treatment was needed or sought. Second, data were coded by trained personnel based on narratives abstracted from patients’ medical records, for which details of the injuries and circumstances varied. Inaccuracies in the abstraction and coding process might have occurred. Third, differences by race and ethnicity could not be examined because of the high prevalence of missing race/ethnicity data (20.3%). Finally, data are based on information in the ED record and are not linked to other data sources (e.g., police reports or school disciplinary reports) that might provide additional information about the circumstances related to the injury or the relationship between the perpetrator and victim.

Although the number of young persons treated for nonfatal assault injuries in EDs is declining, and this trend is promising, these injuries remain common and costly. In 2015, approximately 485,610 young persons were treated for assault-related injuries, and associated medical and lost productivity costs were approximately $3.4 billion (*1*). These injuries continue to occur most often among males and among young adults aged 20–24 years, highlighting the groups that need to be reached with continued and enhanced prevention strategies. Violence among young persons is preventable with the implementation of evidence-based policies and programs that significantly reduce the risk for injuries and associated risk factors. CDC’s *A Comprehensive Technical Package for the Prevention of Youth Violence and Associated Risk Behaviors* (https://www.cdc.gov/violenceprevention/pdf/yv-technicalpackage.pdf) can help states and communities focus their collaborative action on strategies supported by the best available evidence (*2*).

**Summary**

**What is already known about this topic?**

Persons aged 10–24 years account for a substantial proportion of nonfatal assault injuries treated in emergency departments (EDs) in the United States.

**What is added by this report?**

The 2015 rate for nonfatal injuries among persons aged 10–24 years was 753.2 per 100,000 population, the lowest rate in the 15-year study period (2001–2015). From 2011 to 2015, injury rates declined among both males and females and all age groups examined. Despite these findings, assault injuries continue to occur often, with 485,610 young persons treated in EDs for assault-related injuries in 2015.

**What are the implications for public health practice?**

Primary prevention strategies that build communication and problem-solving skills and address risk factors for violence among young persons can stop violence before it starts. Expansion of these strategies and additional interventions focused on injured young persons while they are receiving ED treatment to connect to immediate and ongoing community support might decrease the risk for reinjury or fatality. CDC’s technical package to prevent youth violence helps communities and states prioritize strategies with the best available evidence.

## Footnotes

* https://surveillance.cancer.gov/joinpoint/.
